# A Microneedle Functionalized with Polyethyleneimine and Nanotubes for Highly Sensitive, Label-Free Quantification of DNA

**DOI:** 10.3390/s17081883

**Published:** 2017-08-16

**Authors:** Darius Saadat-Moghaddam, Jong-Hoon Kim

**Affiliations:** School of Engineering and Computer Science, Washington State University, Vancouver, WA 98686, USA; d.saadat-moghaddam@wsu.edu

**Keywords:** amperometric sensors, carbon nanotubes, DNA, polyethyleneimine

## Abstract

The accurate measure of DNA concentration is necessary for many DNA-based biological applications. However, the current methods are limited in terms of sensitivity, reproducibility, human error, and contamination. Here, we present a microneedle functionalized with polyethyleneimine (PEI) and single-walled carbon nanotubes (SWCNTs) for the highly sensitive quantification of DNA. The microneedle was fabricated using ultraviolet (UV) lithography and anisotropic etching, and then functionalized with PEI and SWCNTs through a dip coating process. The electrical characteristics of the microneedle change with the accumulation of DNA on the surface. Current-voltage measurements in deionized water were conducted to study these changes in the electrical properties of the sensor. The sensitivity test found the signal to be discernable from the noise level down to 100 attomolar (aM), demonstrating higher sensitivity than currently available UV fluorescence and UV absorbance based methods. A microneedle without any surface modification only had a 100 femtomolar (fM) sensitivity. All measurement results were consistent with fluorescence microscopy.

## 1. Introduction

To increase DNA testing quality and avoid the unnecessary use of limited amounts of DNA from samples, a reliable estimate of the DNA concentration is crucial for ensuring that proper quantities of DNA are being used for downstream analysis [1]. DNA quantification is necessary for numerous biological applications, ranging from traditional molecular biological manipulations such as restriction digest analysis, Southern blotting, short tandem repeat (STR) analysis and polymerase chain reaction (PCR) [2] to diagnostic techniques, such as the quantification of genetically modified organism (GMO) content of samples [3], detection of DNA contamination in drug preparations produced from recombinant organisms, and medical diagnosis of virus and cancer [4,5]. The quantification and characterization of DNA is often regarded as a labor intensive process in bioscience laboratories. Thus, simple and reliable methods to determine the precise concentration of DNA are much needed for high-quality DNA analysis. Currently, the most common technique to determine DNA concentration involves measuring the absorbance of UV light at 260 nm. Although a relative simple and rapid method, the UV absorbance suffers from low sensitivity and fails to distinguish double stranded DNA (dsDNA) from single-stranded DNA (ssDNA) and RNA [6]. PCR can be used to estimate the concentration of DNA. However, PCR is slow, expensive, and requires complex sample preparation steps. Fluorescence based methods are also used to measure the concentration of DNA [5], yet are vulnerable to contaminants binding with probes and eliciting false results [6]. Additionally, fluorescence particles may degrade during measurement, possibly causing fluctuation in the results.

To develop a rapid, simple, and reliable sensing method, a substantial number of studies have been performed on nanoscale components with the purpose of innovating sensors [7]. Among the various nanomaterials, carbon nanotubes (CNTs) have received considerable attention as additives to enhance the sensitivity and electron transfer kinetics of electrodes [8,9]. CNT-based electrodes display fast electron transfer kinetics due to the sp2 hybridized CNT structure being highly conductive and the ends of CNTs having reactive edge plane sites. In addition, CNTs are especially attractive for smaller electrodes because their high surface-area-to-volume ratio provides a large electroactive surface available for the adsorption of biomolecules. However, a significant challenge associated with the use of CNTs involves breaking apart aggregates to achieve a monodispersion [10]. 

**Figure 1:**
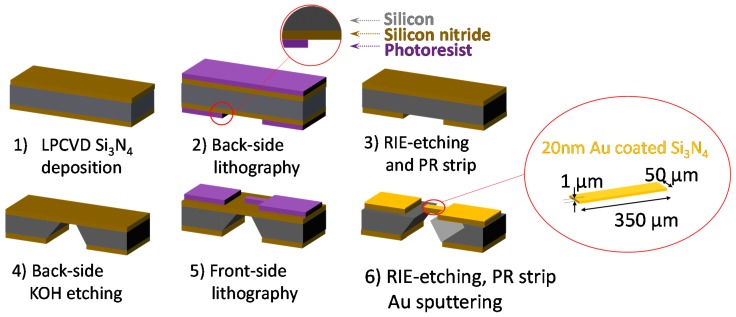
Microneedle fabrication process: (1) Low-Pressure Chemical Vapor Deposition (LPCVD) 1 μm-thick low-stress silicon nitride (Si_3_N_4_) film deposition; (2) Photolithography patterning on the back side; (3) Reactive ion etching (RIE) to open the back side and photoresist (PR) strip; (4) Anisotropic undercut etching of Si with potassium hydroxide (KOH); (5) Photolithography on front side; (6) RIE to define microneedle, PR strip, and 20 nm-thick gold (Au) sputtering for electrical conduction.

In this paper, a well-developed top-down microfabrication method (Figure 1) was combined with a bottom-up nanotubes assembly to produce a functionalized microneedle sensor at high yield. After fabrication, polyethylenimine (PEI), which is known to effectively interact with CNTs via physiorption on the CNTs’ sidewalls, was then coated on the microneedle surface by a dip coating process [4]. The high affinity of PEI for single walled carbon nanotubes (SWCNTs) led to its use as an adhesive layer for fouling-free surface. Lastly, SWCNTs were coated as a means of enhancing the sensitivity. In order to obtain uniform dispersion, this study employed sonication for the dissolution of CNTs in a dimethylformamide (DMF) solution. An alternating current (AC) field was used to concentrate DNAs onto the functionalized microneedle sensor. Electrical measurement was then performed in deionized (DI) water to quantify DNA concentrations. DI water was chosen so as to reduce chemical side-reactions, thus enabling a reference-free detection. The combination of PEI, SWCNTs, AC facilitated capture, and amperometry proved fruitful as evidenced by the sensitivity test, during which there was clear differentiation in the data when using λ DNA concentrations as low as 100 attomolar (aM). The aforementioned results, supported by fluorescence microscopy using an intercalating dye, represents a 1000 fold sensitivity improvement relative to current methods based on UV spectroscopy [11]. The advantages of a label-free quantification approach includes less resources for assay development, minimized sample processing, and direct real-time information on analyte binding [12].

## 2. Materials and Methods

### 2.1. Microneedle Fabrication

A conventional microfabrication technique was employed for a reproducible, high-throughput, and low-cost microneedle sensor. As illustrated in Figure 1, the following steps were taken to fabricate the microneedle: (1) 1 μm thick low stress silicon nitride (Si_3_N_4_) film was formed by low-pressure chemical vapor deposition (LPCVD); (2) photolithography patterning on the back side; (3) reactive ion etching (RIE) of Si_3_N_4_ film to open the back side for wet etching; (4) anisotropic undercut etching of Si with potassium hydroxide (KOH) to make a free-standing cantilever structure (1 μm in thickness); (5) photolithography on front side of wafer to define the microneedle; (6) RIE to complete microneedle pattern and deposition of 10 nm-thick chrome (Cr) then 20 nm-thick gold (Au) layer via sputtering. Chrome was added to act as an adhesion layer between the gold layer and Si_3_N_4_.

### 2.2. Surface Functionalization and Characterization

The microneedle was functionalized with PEI and SWCNTs using a dip-coating method. Dip-coating is one of the easiest and fastest methods to prepare thin films from solutions with the highest degree of control. The working principle is as simple as dipping the substrate into a solution and then withdrawing it at a constant speed. When pulling the substrate upward at a constant speed, the solution is homogeneously entrained on the substrate by the combination of viscous drag and capillary rise. Evaporation then takes over and leads to solidification of the final coating. All of the following surface modification and experimental procedures were conducted in a controlled environment at room temperature. In this paper, the viscosity of the solution was varied to control the film characteristics, while maintaining the withdrawal speed. To find out the optimal concentration of PEI for the uniform coating, PEI (50% *w/v* in H_2_O, Sigma-Aldrich, St. Louis, MO, USA) was prepared in DI water at the concentration of 0.5%, 1%, and 2%. The purified SWCNT bundles were dispersed in DMF (100 mg/L) by ultrasonication for 4 h. When bulk SWCNTs are utilized, the charge transport through SWCNTs is averaged over metallic and semiconducting tubes [13]. The electron transfer characteristics through the radial direction of SWCNTs are similar for metallic and semiconducting tubes [14], which is advantageous for reproducible measurement regardless of chirality of SWCNTs. 

The microneedle was mounted onto an automated stage driven by a linear motor, thus enabling precise control for the PEI-SWCNT surface modification First, the microneedle was immersed into PEI solution for 5 min and withdrawn at a constant velocity of 2 mm/s. PEI was used as an adhesive layer due to their strong interaction with CNTs. PEI-coated microneedle was then cured at 150 °C in a furnace for 10 min. Subsequently, the PEI-coated microneedle was re-mounted onto the stage and immersed into a SWCNT-DMF suspension for 5 min and withdrawn at a constant 2 mm/s velocity. Through the dip coating process, a PEI and SWCNT layer was obtained on the microneedle surface. Fourier transform infrared spectroscopy (FTIR) and surface profilometry were also used to characterize the PEI and PEI/SWCNTs layers, results can be found in the Supplementary Materials Section (Figures S1 and S2). 

### 2.3. Electrical Measurement

Amperometric sensing is most useful when the analyte binding significantly perturbs the sensing interface [14]. The current-voltage (IV) measurement was conducted in DI water using a picoammeter (Keithley 6487 Picoammeter/Voltage source, Tektronix, Inc, Beaverton, OR, USA). IV measurements were used to quantify DNA concentration as well as assess the quality of the functionalization layers when using different concentrations of PEI. DI was chosen since conventional electrochemical buffers are susceptible to temperature- and humidity-induced changes in ionic concentration, thus necessitating calibration by a reference electrode and adding undesirable complexity [15,16]. Using DI water for the IV measurement reduces the redox potential, leading to an increase in the signal-to-noise ratio, and enabling measurement of electrical properties without a reference electrode [17]. The procedure for the IV measurement is as follows: the microneedle sensor was immersed in 5 μL of DI water using a motorized stage; a voltage was swept from 0 to 1.4 V and the electrical current was measured between the sensor (working electrode) and the circular stainless sample well (counter electrode). The distance between the two electrodes is about 1.5 mm.

**Figure 2:**
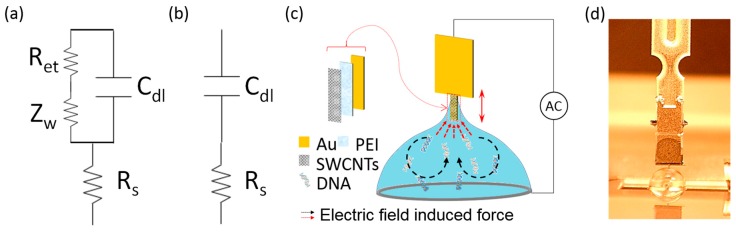
(**a**) General equivalent circuit for electrochemical measurement; (**b**) Equivalent circuit for electrochemical measurement in the absence of the redox probe; (**c**) Working principle of a microneedle: DNA in a sample is concentrated by electrokinetic flow (black arrow). The concentrated DNA is further attracted to the microneedle by dielectrophoresis (red arrow). The attracted DNA is captured on the microneedle surface with capillary action when the microneedle is withdrawn from the solution. The captured DNA is detected in DI water through the electrical measurement; (**d**) Picture of the microneedle immersed in the sample solution: experimental setup composed of the functionalized needle, a solution drop in a well plate, a signal generator, and a picoammeter.

The equivalent circuits for the electrochemical measurement can be seen in Figure 2a, which is composed of the ohmic resistance of the electrolyte solution (R_s_), the Warburg impedance (Z_w_) resulting from the diffusion of ions from the bulk electrolyte to the electrode interface, the double layer capacitance (C_dl_), and electron transfer resistance (R_et_) that exists if a redox probe is present in the electrolyte solution. Due to the suppression of redox reaction in DI water, the characterizing parameters for electron transfer, R_et_ and Z_w_, become infinite, and the equivalent circuit can be simplified to Figure 2b [18]. Since R_s_ represents bulk properties of the electrolyte solution, it is not affected by chemical transformations occurring at the electrode surface. Therefore, the build-up of the sensing analyte on the electrode surface mainly alters the double layer capacitance, C_dl_. Therefore, cyclic voltammetry (PARSTAT 4000 potentiometer, AMTEK Princeton Applied Research, Oak Ridge, TN, USA) was also performed in DI water with bare gold, 1% PEI-coated, PEI/SWCNTs-coated, and functionalized microneedles with captured DNA to illustrate a change in the double layer capacitance. 

### 2.4. Sensitivity Test

In order to check sensitivity of the functionalized microneedle, λ-DNA (48.5 kbp, 31.5 kDa, New England Biolabs, Ipswich, MA, USA) was used as a model analyte. The λ-DNA was suspended in 1× Tris EDTA (TE) buffer with a pH of 7.5 and diluted using the same buffer to prepare sample solutions with concentrations ranging from 100 aM to 1 pM in 10-fold increments. To control the sample volume precisely, a pipette (Eppendorf Research plus adjustable volume pipette) was utilized to dispense a consistent volume of solution for all the experiments. For the concentration, an AC field (5 MHz, 20 V_peak-to-peak_) was applied for 1 min while the functionalized microneedle was immersed in a 10 μL solution containing λ-DNA at different concentrations. As illustrated in Figure 2c, the application of the AC field concentrates target analytes by electrokinetic flow. Targets are subsequently polarized causing a dipole moment, further attracting analytes to the needle’s surface (dielectrophoresis). After conducting the capture step described in the previous section, the sensitivity of the sensor was assessed by taking an electrical measurement at various concentrations of λ-DNA. The control experiment involved substituting the solution used in the DNA capture step with 10 μL of pure TE buffer. All other parameters, including the application of the AC field, were unchanged. Figure 2d shows a picture of the microneedle immersed in the DNA sample solution. The sensitivity test was also performed with bare microneedles without surface modification for comparison. The experimental setup for sensitivity test composed of a functionalized microneedle, a solution drop in a stainless well, and a signal generator. For all the experiments, the axial motion of the microneedle sensor was controlled by a motorized stage in order to maintain a consistent distance between the terminal end of the microneedle and the well. All experiments were iterated at least 3 times. To validate the electrical measurement, a green intercalating dye (PicoGreen^®^, excitation and emission wavelengths: 480 nm and 520 nm, Thermo Fisher Scientific, Waltham, MA, USA) was utilized for the visualization of surface-bound λ-DNA. After a 200-fold dilution with 1× TE buffer, the DNA captured microneedle was incubated in 5 μL of the diluted PicoGreen for 5 min at room temperature The sensor was then imaged by a fluorescence microscope (Nikon Eclipse CI-S, Nikon Instruments Inc., Melville, NY, USA). ImageJ (National Institutes of Health, Bethesda, MD, USA) software was used to quantify the fluorescence intensities.

## 3. Results and Discussions

**Figure 3:**
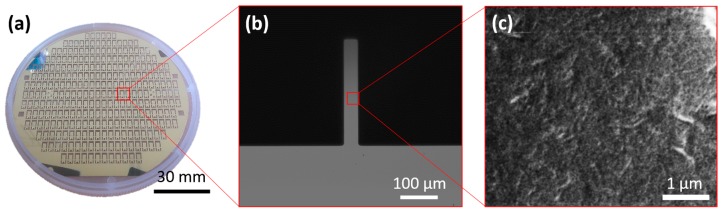
Scalability of the microneedle fabrication. (**a**) Two hundred eighty microneedles in a 100-mm wafer; (**b**) Picture of a microneedle; (**c**) Surface of microneedle uniformly coated with PEI and SWCNTs.

The scalability in manufacturing of microneedles was demonstrated using microfabrication as shown in Figure 3a. Figure 3b presents an optical image of a microneedle, with dimensions of 50 μm wide and 350 μm long. Two hundred and eighty microneedles can be manufactured on a 100 mm-diameter Si wafer. Typically, 25~50 wafers are processed in one batch, which can significantly reduce the costs associated with fabrication and assay preparation. The uniform shape of the microneedle is also crucial in controlling background noise because the non-uniform geometry of microneedle could increase the nonspecific capture, which consequently increases the background noises. The fabricated microneedles were then functionalized with PEI and SWCNTs through the dip-coating process to enhance the sensitivity. Figure 3c shows a SEM image of the PEI/SWCNTs uniformly coated on the surface [19].

**Figure 4:**
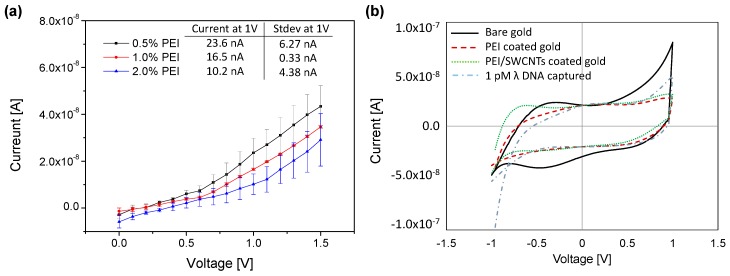
(**a**) IV curve for the SWCNTs coated microneedle with various concentration of PEI; (**b**) Cyclic voltammetry for four different types of surfaces: bare gold, 1% PEI-coated, 1% PEI/SWCNTs-coated microneedle, and 1 pM λ DNA captured needle.

In order to assess the quality of the functionalization layers with different concentration of PEI, the IV measurement was conducted using a picoammeter. Three different concentrations of PEI were tested at a constant withdrawal speed of 2 mm/s for the surface coating: 0.5%, 1%, and 2% PEI. Concentrations higher than 2% were not tested due to inconsistencies in the coating layer. As shown in Figure 4a, the average currents with standard deviations at 1 V were 23.6 ± 6.27, 16.5 ± 0.33, and 10.2 ± 4.38 nA for the SWCNTs coated microneedle with 0.5%, 1% and 2% PEI, respectively. Thus, the higher concentration of PEI provided lower current values with 1% PEI demonstrating the greatest stability in its electrical response. This result indicated that the 1% PEI was the optimal concentration for achieving a uniform coating when using a withdrawal speed of 2 mm/s. As a result, 1% PEI was chosen for cyclic voltammetry and sensitivity tests. 

Figure 4b shows the cyclic voltammetry characteristics for the microneedle throughout the stepwise functionalization of PEI, SWCNTs, and λ DNA. The CV measurement was conducted individually for each layer. In the graph, the inner region of cyclic voltammetry for one cycle represents the capacitance, which is accumulated charge on the microneedle surface. The capacitance decreased with introduction of 1% PEI as compared to the Au electrode, while it increased again when the SWCNTs was bound onto the PEI layer. When DNA was captured on the surface, it was slightly decreased. This indicates that capacitance has some form of dependence on the coating layer. 

**Figure 5:**
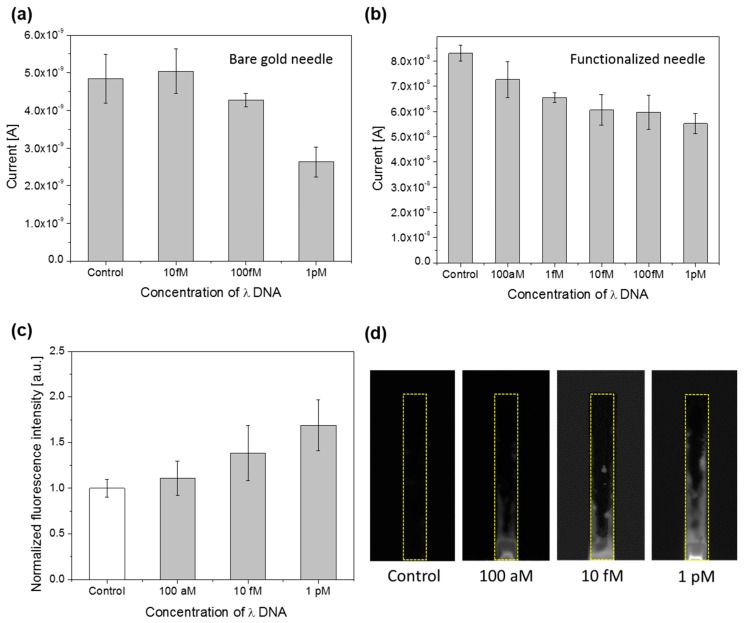
Electrical measurement results, with error bars showing standard deviation, for (**a**) the bare microneedle and (**b**) the functionalized needle at 1.1 V. Upon presence of DNA on the microneedle sensor surface, the current is decreased; (**c**) Fluorescence measurement results with bars showing standard deviation, for the functionalized microneedle. Fluorescence signal increased with increasing DNA concentrations; (**d**) Fluorescence images for control, 100 aM, 10 fM, and 1 pM.

For the sensitivity test, various concentrations of λ DNA were captured and detected on both bare and functionalized microneedles. The results of the sensitivity tests are presented in Figure 5a–c. While the sensitivity using bare microneedles was 100 fM (3.2 ng/mL), the sensitivity when using functionalized microneedles was 100 aM (3.2 pg/mL) with an assay time of 5 min for the electrical measurement. In the electrical measurement, the current would drop with increasing DNA concentrations; indicating charge transfer across the SWCNT surface declined as DNA hybridized. The PEI/SWCNT functionalized needle also had a larger ΔI. The ΔI between the control and 1 pM signal for the functionalized microneedle was 27.9 nA, while the ΔI for the bare gold needle was only 2.22 nA. The results indicate that the SWCNT network enhanced the signal response by 10-fold. In terms of sensitivity, the functionalized microneedle represents a 1000-fold improvement over the bare gold needle. The sensing element consists of SWCNTs on top of a PEI layer [20], enhancing changes in electric signals by inducing a deformation of the electronic structures. Thus, when a target DNA binds to the PEI/SWCNTs, the electric current between the microneedle and well-electrode was changed due to modifications in the electrical properties of SWCNTs and the SWCNT–liquid interface. In addition, the SWCNTs may have enlarged the surface area of sensor, thereby imparting a higher sensitivity towards DNA. 

To support the results of the electrical measurement, as well as verify the existence of DNA on the surface of the functionalized microneedle, a fluorescence measurement was also conducted. With higher concentration of DNA, the fluorescence intensity increased due to more binding events with intercalating dye. As can be seen in Figure 5c,d, while fluorescence measurement demonstrated the presence of DNA on the surface, the error bars become overlapped at the low concentration. 

This detection method is highly sensitive, partially because SWCNTs are used as a sensing element, but also because of the two phenomena occurring during the active concentration step: (1) electrohydrodynamic flow and (2) dielectrophoresis. Therefore, a microneedle sensing platform has demonstrated great potential as an inexpensive and sensitive biosensor. The efficacy of the concentration can be improved by further optimization of an electric field and fluid flow. To confer the specificity, the surface of microneedle can be functionalized with sequence specific probes. However, sequence specific detection of DNA requires a more rigorous experimental setup with a refined experimental protocol. The performance of the microneedle has been summarized in Table 1 and is juxtaposed with commonly used commercial methods. 

## 4. Conclusions

In summary, a highly sensitive and simple method for the measurement of DNA concentration was developed using microneedles functionalized with PEI and SWCNTs. Using the dip-coating method, a uniform coating of PEI and SWCNTs was achieved. Additionally, small geometries and confined current paths allow the electrical properties of the microneedle to be altered with the binding of DNA. These changes in electrical properties can then be quantified with a current measurement and correlated to specific concentrations of target DNAs. Since electrical measurements were conducted through a medium of DI water, minimizing extraneous chemical reactions, the electric measurement setup did not require a reference electrode. The detection results were also validated though fluorescence microscopy. The experimental setup adopted in this study was able to achieve a sensitivity of 100 aM with an assay time of 5 min. The simple measurement configuration will offer a convenient means to rapidly quantify DNA for further analysis.

## Figures and Tables

**Table 1:** Comparison between our functionalized microneedles and the conventional methods [21].

Method	Sensitivity	Label-Free	Processing Time	Sample Volume
Funtionalized microneedle	3.2 fg/μL	Yes	2 min	5~10 μL
UV spectrophotometer	2 ng/μL	Yes	30 s	0.5~2 μL
Fluorometer	10 pg/μL	No	5 min 20 s	1~20 μL

## Supplementary Materials

The following are available online at http://www.mdpi.com/1424-8220/17/8/1883/s1, Figure S1: FT-IR spectrum for PEI/SWCNTs coated microneedle, SWCNTs in DMF, and 1% PEI. Results indicate the presence of the coating layers on the microneedle’s surface, Figure S2: Thickness and surface roughness of (a) PEI coated and (b) PEI/SWCNTs coated microneedles.
