# Supplementary material for: A Microneedle Functionalized with Polyethyleneimine and Nanotubes for Highly Sensitive, Label-Free Quantification of DNA
Source: Sensors (Basel). 2017 Aug 16;17(8):1883. doi: 10.3390/s17081883 (PMC5579740; doi:10.3390/s17081883)
Supplement: Supplementary file 1 [file sensors-17-01883-s001.pdf]

Supplementary information

A Microneedle Functionalized with Polyethyleneimine and Nanotubes for highly sensitive, label free quantification of DNA

Darius Saadat-Moghaddam[1] and Jong-Hoon Kim[1*]

1. School of Engineering and Computer Science, Washington State University, Vancouver, WA, 98686

*Correspondence and requests for materials should be addressed to J.-H.K. (jh.kim@wsu.edu)

Supplementary Note 1: Characterization of functionalization layers using ATR-FTIR

The functionalization of the microneedle was confirmed by Attenuated total reflection-Fourier transform infrared spectroscopy (ATR-FTIR). FTIR is a useful analysis tool using mid-infrared radiation with wavenumber in the range of 4000 and 670 cm^{-1}. In this study, FTIR was used to qualitatively examine the coating layers on the microneedle. FTIR spectra were obtained through the ATR method using a Nicolet iS5 FTIR (ThermoFisher) equipped with a single reflectance apparatus and a diamond prism as an internal reflection element. 1% PEI in DI water, SWCNTs in DMF, and a PEI/SWCNTs coated microneedle were qualitatively tested and compared. As shown in Fig. S1, the IR spectra for 1% PEI in DI water, SWCNTs in DMF, and PEI/SWCNTs coated microneedle verified the presence of coating layers.

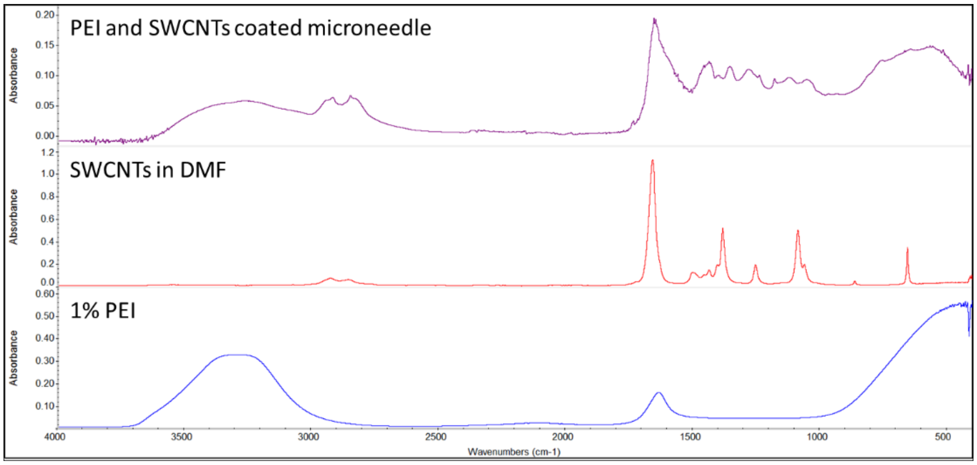

Figure S1. FT-IR spectrum for PEI/SWCNTs coated microneedle, SWCNTs in DMF, and 1% PEI. Results indicate the presence of the coating layers on the microneedle's surface.

Supplementary Note 2: Thickness of functionalization layer measurement

After the functionalization, the film thickness and roughness were determined by surface profilometry (AlphaStep D-300, KLA Tencor). Fig. S2 shows the surface profile for a microneedle coated with either just PEI or PEI/SWCNTs. The results indicate the thickness of PEI layer and SWCNT layer to be 815 nm and 281 nm respectively. By taking an arithmetic average of the variation across the surface, the roughness of the PEI layer and SWCNT layer was found to be 5.15 nm and 114 nm respectively. Based on the aforementioned results, the introduction of SWCNTs leads to a 20 fold increase in the surface roughness. In addition, multiple layers of SWCNTs must be present on the surface of the microneedle when considering the small diameters of the SWCNTS in conjunction with the measured thickness of the SWCNT layer.

a After PEI coating   b After PEI and SWCNTs coating

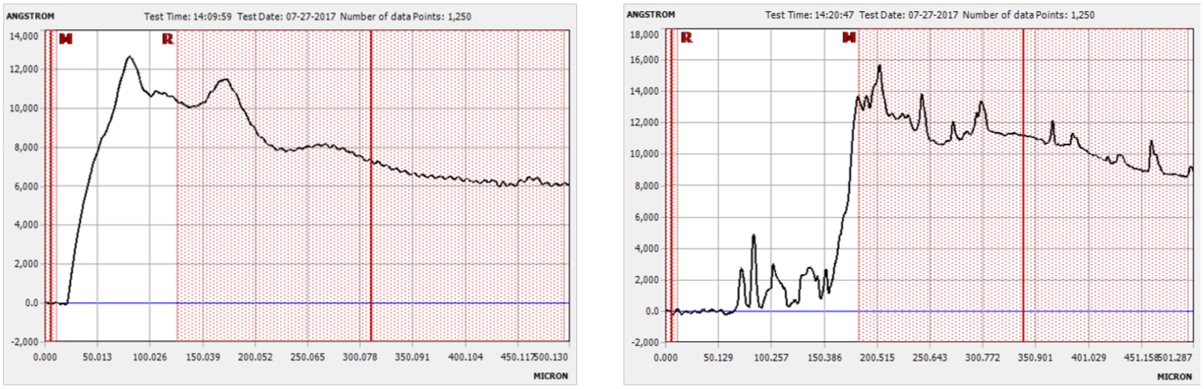

Average coating thickness: 815 nm   Average coating thickness: 1,096 nm

Figure S2. Thickness and surface roughness of (a) PEI coated and (b) PEI/SWCNTs coated microneedles.
